# Variability of T1 in purpose recruited normal volunteers and patients as a function of shim (B0), flip angle (B1) and myocardial sector at 3T

**DOI:** 10.1186/1532-429X-17-S1-P5

**Published:** 2015-02-03

**Authors:** Anupama K Rao, Anders M Greve, Sonia Nielles-Vallespin, Bruce S Spottiswoode, Kelvin Chow, Richard B Thompson, Peter Kellman, Andrew E Arai

**Affiliations:** 1 National Heart, Lung and Blood Institute, National Institutes of Health, Bethesda, MD, USA; 2 Cardiovascular MR R&D, Siemens Medical Solutions, Chicago, IL, USA; 3 Department of Biomedical Engineering, University of Alberta, Edmonton, AB, Canada

## Background

The purpose of this study was to assess inter-subject and spatial variability of T1 measurements in normal volunteers and investigate potential reasons for non-uniform T1 measurements such as off resonance and imperfect flip angle at 3T. A comparison group of patients with normal studies was used to assess how well findings translate to the clinical setting.

## Methods

36 subjects (including 20 normal volunteers) were scanned using an investigational prototype sequence on a 3T scanner (MAGNETOM Skyra, Siemens AG, Germany). T1 was measured using three Methods

MOLLI with a flip angle of 20˚, MOLLI with flip angle 35˚, and 2-parameter SASHA with a variable flip angle (VFA) readout. B0 and B1 field maps were generated. Sector level, intra-subject, between-subject, B0-related, and B1-related variability of T1 was assessed with coefficient of variation (COV) and clustered linear mixed models.

## Results

The mean T1 value for normal volunteers varied by T1 method (Table, pairwise comparisons all p<0.001). Between-subject variation (based on a single average T1 per subject) was about 3% and did not vary significantly between methods (Table). However, intra-subject variability was about 5% for all three methods. Between-subject spatial variation of T1 at a sector level was ±1.5% for both T1 MOLLI flip angle 20˚ and MOLLI flip angle 35˚, and ±1.3% for SASHA.

Using a clustered linear mixed model, absolute B0 offset (shim) was the strongest predictor of T1 for MOLLI flip angle 20˚ (p<0.0001), MOLLI flip angle 35˚ (p<0.0001), and SASHA (p=0.0056). Between-subject effects were weaker but significant for MOLLI flip angle 20˚ (p=0.0001), MOLLI flip angle 35˚ (p=0.0002), and SASHA (p=0.0028). B1 (% prescribed flip angle) was a significant predictor of T1 for all methods in univariable analysis but generally not significant in multivariable analysis. However, we had limited numbers of sectors with a flip angle deviation >20%.

Similar findings were found in patients imaged only with the MOLLI flip angle 20˚. Between-subject variation in T1, intra-subject variation of T1, and B0 offsets were not significantly different from the normal volunteers (Table 1).

**Table 1:** Comparisons of Normal Volunteers and Patients

	Normal Volunteers (N=20)	Patients (N=16)
Mean T1 MOLLI FA 35˚ Between-subject COV (Mean±SD) Intra-subject COV (Maximum)	1247±54 ms 3%±1.2% 5.3%	NA

Mean T1 MOLLI FA 20˚ Between-subject COV (Mean±SD) Intra-subject COV (Maximum)	1297±50 ms 2.6%±1.1% 5.4%	1318±47 ms 2.7%±1% 4.3%

Mean T1 SASHA Between-subject COV (Mean±SD) Intra-subject COV (Maximum)	1539±47 ms 2.6%±0.9% 4.7%	NA

Shim: Mean Per subject Absolute B0 Offset ± SD	41.2±17.4 Hz	34.0±18.7 Hz

Shim: Avg Per Subject Max B0 Offset ± SD	90.7±27.2 Hz	85.6±54.8 Hz

Flip Angle: Mean per subject B1 (% of prescribed)	101.7±5.2 %	NA

Flip Angle: Avg per Subject Max B1 (% of prescribed)	118.2±5.9%	NA

## Conclusions

B0 inhomogeneity is the biggest source of error in measuring T1 at 3T. B0 offsets artifactually reduce measured T1 by MOLLI and to a lesser degree by SASHA. Between-subject effects are generally second order after B0 effects.

## Funding

Funded by the Intramural Research Program of the National Heart, Lung and Blood Institute (NHLBI), National Institutes of Health, Department of Health and Human Services, Bethesda, MD.

